# Novel Compensation Scheme for the Modulation Gain to Suppress the Quantization-Induced Bias in a Fiber Optic Gyroscope

**DOI:** 10.3390/s17040823

**Published:** 2017-04-10

**Authors:** Xiong Pan, Pengcheng Liu, Shaobo Zhang, Jing Jin, Ningfang Song

**Affiliations:** Institute of Opto-electronics Technology, School of Instrument Science and Opto-electronics Engineering, Beihang University, Beijing 100191, China; 08768@buaa.edu.cn (X.P.); zhangshaobo@buaa.edu.cn (S.Z.); jinjing@buaa.edu.cn (J.J.); Songnf@buaa.edu.cn (N.S.)

**Keywords:** fiber optic gyroscope, modulation gain, quantization error, scale factor, bias

## Abstract

A novel digital compensation scheme is demonstrated to control the gain of the modulation chain and suppress the influence of quantization error on bias. The error produced by the quantization multiplied by the scaling factor is theoretically analyzed. Simulations indicate that the quantization error varies with the input angular velocity and temperature, which is verified by experiments. By switching the integration and compression operations in the modulation chain, this quantization error is reduced, while automatic reset of the digital phase ramp register is achieved. We test the scheme in a fiber optic gyroscope. The test results reveal that the quantization-induced bias is suppressed and the residual bias is two times less than the desired accuracy with data accumulated over one-second sample interval. The scheme is a feasible method to miniaturize fiber optic gyroscope using a totally digital circuit for compensation of the modulation gain.

## 1. Introduction

The fiber optic gyroscope (FOG) is widely used in navigation applications [1]. In an all-digital closed-loop FOG, the electro-optic coefficient of the integrated optic modulator (IOM) varies with temperature. Hence, the gain of the modulation chain drifts, which affects the scale factor stability [2]. Meanwhile, when the digital phase ramp resets, the height of the phase reset is no longer equal to 2π radian, and the imperfect 2π reset deteriorates bias and noise [3,4,5]. Conventionally, the gain drift is compensated by a second feedback loop using another D/A converter (DAC), through which the reference voltage of the DAC in the modulation chain is adjusted [2,3,4,6,7]. This analog compensation method achieves high scale-factor stability without deterioration of bias and noise, but it needs more complex electronics, which means extra volume and power consumption.

To simplify the circuit, a digital compensation scheme has been put forward by integrating the function of the second feedback loop into a digital logic chip [8]. Ma et al. focused on the verification of the effect of using a digital compensation procedure for replacing the classical second feedback loop on the scale factor stability. However, the quantization errors, which might induce unstable spurious bias, were not discussed. At that time, although the spurious bias deviated from the normal value, the bias repeatability at a fixed temperature met the performance requirements, and the variation of spurious bias with scaling factor (in fact, temperature induced variation of 2π reset voltage) was compensated by temperature compensation procedure mainly for suppressing the Shup error in fiber coil. Latterly, we compensated the spurious bias by adding the truncated part of the products of scaling factor and digital phase step onto the output of FOG based on the digital algorithm in [8]. Besides, the algorithmic implementation is complicated because of the register operation of the forced reset.

In this paper, we present the improved digital compensation method, which depressed the unstable spurious bias by differential operation of the optical path and time-average, without any additional procedures. We believe it is time to discuss the quantization errors of both kinds of digital compensation algorithms. The quantization errors on the bias are theoretically analyzed based on the simplified model of the digital close-loop FOG. Next, the suppression of the quantization error is demonstrated in the improved scheme, which leaves the auto-reset of the digital phase ramp known as one of the advantages of all digital close-loop FOG. Simulations are carried out to investigate in detail the bias of the two digital compensation schemes at different input angular velocities and digital scaling factors. Finally, we validate the improved scheme by comparing the performance of the two types of digital schemes with that of the conventional analog method on the same FOG.

## 2. Analysis of the Compensation for the Gain Drift of the Modulation Chain

**Figure 1:**
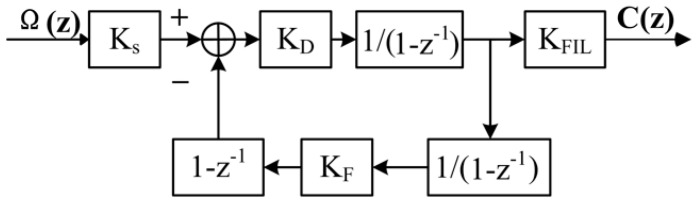
The simplified linearized model of an all-digital closed-loop fiber optic gyroscope (FOG). Ω: the input angular velocity, C : the output data of the FOG, $K_{s}$ : the proportional coefficient of the Sagnac Effect, $K_{D}$ : the total gain of the detection chain, $K_{FIL}$ : the gain of the digital filter in the output chain, and $K_{F}$ : the total gain of the modulation chain, $1/\left(1-z^{-1}\right)$ : the integration operation, $\left(1-z^{-1}\right)$ : the differential operation for the delay between two counter-propagating light waves.

According to the implementation of an all-digital closed-loop FOG, the linearized model is simplified by ignoring the dynamic or transient state [9,10,11,12], as shown in Figure 1.

In view of the configuration of the modulation chain and the digital phase ramp reset, the height of the phase reset $\phi_{r}$ should be 2π radians when the dynamic range width $D_{r}$ of the ramp register is $2^{n}$ (n is the bit length of the register). The modulation gain $K_{F}$ is [2]:

(1) $$K_{F}=\frac{\phi_{r}}{D_{r}}=\frac{2^{n}*K_{DA}K_{A}K_{IOM}}{2^{n}}=\frac{2\pi }{2^{n}}$$

where $K_{DA}$. is the DAC coefficient, $K_{A}$ is the gain of buffer amplifier in the modulation chain, and $K_{IOM}$ is the electro-optic coefficient of the IOM.

In Equation (1), not only should the ratio (modulation gain $K_{F}$) be kept constant to ensure the scale factor stability, but the numerator (the height of the phase reset $\phi_{r}$) should also be exactly 2π radians to avoid the deterioration of the bias and noise. In the conventional analog method, the gain drift is compensated by adjusting the DAC coefficient $K_{DA}$ to counteract the change of the modulator coefficient $K_{IOM}$, and the mentioned requirements of the ratio and the numerator are satisfied simultaneously.

**Figure 2:**
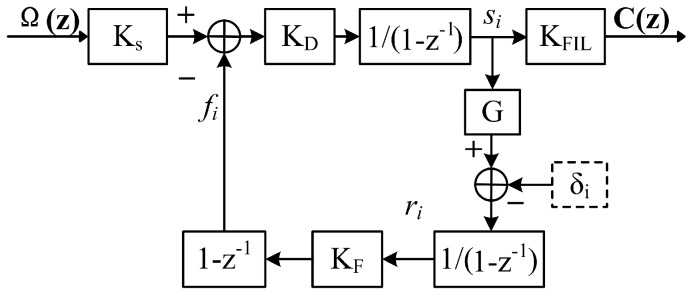
The model of the First-Compression-Last-Integration (FCLI) scheme.

However, when the function of gain compensation is integrated into a digital logic chip, the analog coefficient $K_{DA}$ and $K_{A}$ in Equation (1) can no longer be regulated. Besides, the dynamic range width $D_{r}$ of the ramp register is adjusted to $2^{x}$ to guarantee that the height of the phase reset equals 2π radians. Synchronously, a scaling factor G is introduced to compensate for the change of the modulation gain, as shown in Figure 2. In [8], this scheme is adopted, and we refer to it as the First-Compression-Last-Integration (FCLI) scheme.

It is easy to determine that the scaling factor G equals $2^{x}/2^{n}$, and the compensated modulation gain $K\prime_{F}$ is given by:

(2) $$K\prime_{F}=K_{F}*G=\frac{2^{x}*K_{DA}K_{A}K_{IOM}}{2^{x}}*\frac{2^{x}}{2^{n}}=\frac{2\pi }{2^{n}}$$

When the phase step $s_{i}$ is compressed by multiplying the scaling factor, the quantization error $\delta_{i}$ is induced by the truncation operation due to the finite word-length of the register [13]. The compressed phase step is:

(3) $$\lfloor G*s_{i}\rfloor =G*s_{i}-\delta_{i}=G*\left(s_{i}-\delta_{i}/G\right)$$

where ⌊ ⌋ represents the truncation operation. Then, the compressed phase step is integrated to generate the phase ramp $r_{i}$, and:

(4) $$r_{i}=r_{i-1}+G*\left(s_{i}-\delta_{i}/G\right)$$

After the differential operation, the time-average of the feedback phase shift $f_{i}$ is:

(5) $$\langle f_{i}\rangle =\langle K_{F}*\left(r_{i}-r_{i-1}\right)\rangle =K\prime_{F}*\langle s_{i}-\delta_{i}/G\rangle =K\prime_{F}\langle s_{i}\rangle -K\prime_{F}\langle \delta_{i}/G\rangle$$

where 〈 〉 represents the calculation operation of time-average.

In Equation (5), the time-average of the feedback phase shift $\langle f_{i}\rangle$ is strictly proportional to $\langle s_{i}-\delta_{i}/G\rangle$ rather than the time-average of the phase step $\langle s_{i}\rangle$, and it is equivalent to induce the error $\langle \delta_{i}/G\rangle$ in the phase step of the output chain.

The equivalent bias $\Omega_{bias}$ of angular velocity is:

(6) $$\Omega_{bias}=\frac{K{\text{′}}_{F}}{K_{s}}\langle \frac{\delta_{i}}{G}\rangle$$

According to Equation (6), the equivalent bias varies with both the scaling factor G and the quantization error $\delta_{i}$, which are affected by temperature and input angular velocity, respectively.

As for the FCLI algorithm implemented according to [8], the purpose of the second feedback loop is to obtain the dynamic range width $2^{x}$. Then, the real value of the digital phase ramp register is set by forced reset. The forced reset under various operating conditions makes the FCLI scheme more complicated than the conventional analog scheme, in which automatic reset is adopted.

## 3. First-Integration-Last-Compression Scheme

**Figure 3:**
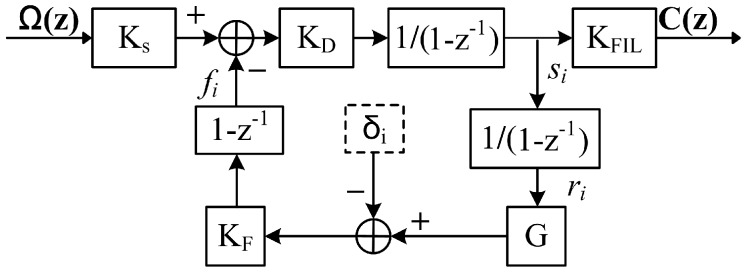
The model of the First-Integration-Last-Compression (FILC) scheme.

In this paper, the scaling factor G is moved after the integrator of the modulation chain, which is equivalent to G in front of the integrator in consideration of the total gain of the modulation chain, as shown in Figure 3. We name this scheme as the First-Integration-Last-Compression (FILC) scheme.

After integration, compression and differential operation, the feedback phase shift is deduced, which is similar to Equations (4) and (5):

(7) $$r_{i}=r_{i-1}+s_{i}$$

(8) $$\lfloor G*r_{i}\rfloor =G*r_{i}-\delta_{i}$$

(9) $$\begin{array}{c} f_{i}=K_{F}*\left(\left\lfloor G*r_{i}\rfloor -\lfloor G*r_{i-1}\right\rfloor \right)=K_{F}*\left\{\left(G*r_{i}-\delta_{i}\right)-\left(G*r_{i-1}-\delta_{i-1}\right)\right\}\\ =K_{F}*\left\{G*\left(r_{i}-r_{i-1}\right)-\left(\delta_{i}-\delta_{i-1}\right)\right\}=K_{F}*\left\{G*s_{i}-\left(\delta_{i}-\delta_{i-1}\right)\right\} \end{array}$$

As for the current feedback phase shift, the FILC scheme still has the quantization error $\left(\delta_{i-1}-\delta_{i}\right)$, but this error is suppressed due to the time-averaging process [2]:

(10) $$\langle \delta_{i-1}-\delta_{i}\rangle =\frac{\sum_{i=2}^{+\infty }\left(\delta_{i-1}-\delta_{i}\right)}{i}\approx 0$$

And:

(11) $$\langle f_{i}\rangle =K\prime_{F}*\langle s_{i}\rangle$$

The time-averaged feedback phase shift is strictly proportional to the time-averaged output phase step in the FILC scheme. That is to say, the phase step is uniform between the modulation chain and the output chain. The quantization error is suppressed by the differential operation of the optical path without any additional digital signal processes.

**Figure 4:**
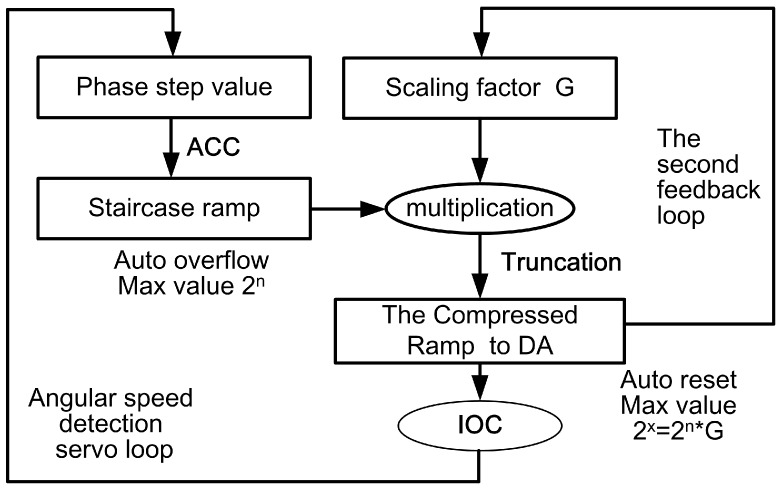
The algorithmic flow-chart of the FILC scheme.

**Figure 5:**
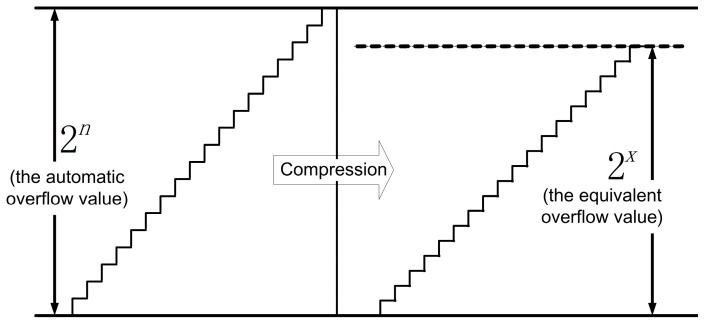
The compressed phase ramp of the FILC scheme.

The algorithmic flow-chart of the FILC scheme is presented in Figure 4. The purpose of the second feedback loop is set to demodulate the scaling factor G directly. The digital phase ramp is reset by automatic overflow with a dynamic range width of $2^{n}$. After compression, the equivalent dynamic range width is transformed to ${D_{r}}^{\prime }=G*2^{n}=2^{x},$ as shown in Figure 5. The requirements for the modulation gain and the height of the phase reset are satisfied simultaneously.

In summary, the improved FILC scheme not only removes the unstable parasitic bias in the original FCLI scheme but also retains the automatic reset, as the conventional analog method does, which simplifies the algorithm implementation. It is a reliable optimized method that integrates the function of gain-control into a digital signal processor, such as Field Programmable Gate Array (FPGA), to miniaturize the FOG.

## 4. Simulation and Experiment

In view of the gain compensation of the modulation loop, the two kinds of digital schemes are equivalent and the scaling factors have the same value. The dynamic performance of the digital compensation scheme has been verified by testing whether or not the scaling factor could vary with temperature in real time and, at the same time, the compensation resolution could be ensured [8]. The dynamic performance is not affected by switching the integration and compression operations. In this paper, we focus on the differences in bias resulting from these two kinds of methods.

**Figure 6:**
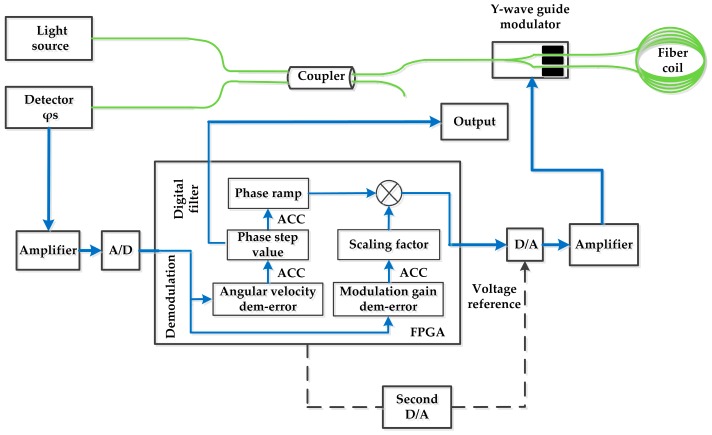
The scheme of the FOG under testing.

The designed parameters of the FOG under testing are presented in Table 1, and the desired accuracy is 0.3°/h with data accumulated over a 1 s sample interval. The schematic diagram of the FOG is shown in Figure 6. The second D/A generates the reference voltage of the first D/A. In the analog scheme, the reference voltage of the first D/A is changed to compensate for the fluctuation of the modulation chain gain caused by varied temperature. Conversely, in the FCLI or FILC scheme, the reference voltage is set to a fixed value while the modulation chain gain is compensated by digital procedure. In Figure 6, the parts denoted by dotted lines are only required in analog scheme. The functional modular and data flow of FILC scheme in the FPGA are also shown in Figure 6.

**Figure 7:**
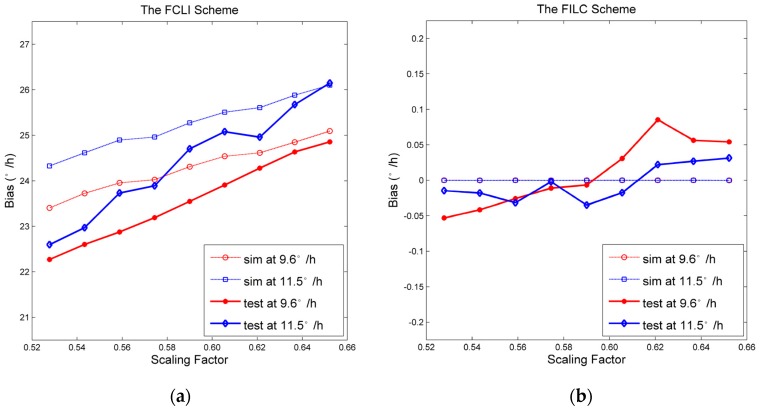
The bias of the FCLI (**a**) and FILC (**b**) schemes.

According to the analysis in Section 2, we know that the quantization-induced bias is affected by the scaling factor and the input angular velocity. In the simulation and experiment, two different angular velocities (9.6°/h and 11.5°/h) are used as the input according to the axis of the FOG pointing to the sky and the north, respectively, in our laboratory located at 40 degrees north latitude. The scaling factor ranges from 0.52 at −40 °C to 0.66 at +60 °C. The bias is simulated according to Equations (6) and (10), as well as tested to evaluate the influences of the quantization error of the FCLI and FILC schemes. The quantization-induced bias is shown in Figure 7. In Figure 7a, the differences between the simulations and experiments of the FCLI scheme can be summarized as the differences of the slope and the intercept of the curves. According to the Equation (6), the quantization error is affected by the Sagnac effect coefficient Ks. Moreover, the intercept of the curves is sensitive to the gain and the noise of the detection chain. It is difficult to measure precisely the $K_{s}$, the gain of detection chain and the noise of the FOG under test. Therefore, these differences between simulation and experiments in Figure 7a are caused by the fact that the parameters in the simulation are not equal to those in the experiment exactly. In Figure 7b, the simulated spurious bias of the improved scheme induced by compression is suppressed to almost zero. Hence the simulations do not vary with $K_{s}$, gain and noise in the detection chain. The differences between the simulations and experiments are caused by the bias repeatability of the FOG under test.

In Figure 7, the quantization-induced bias of the FCLI scheme is unstable, and the variation scale is nine times larger than the desired accuracy. However, the maximum residual bias of the FILC scheme is 0.08°/h, which is two times smaller than the desired accuracy.

**Figure 8:**
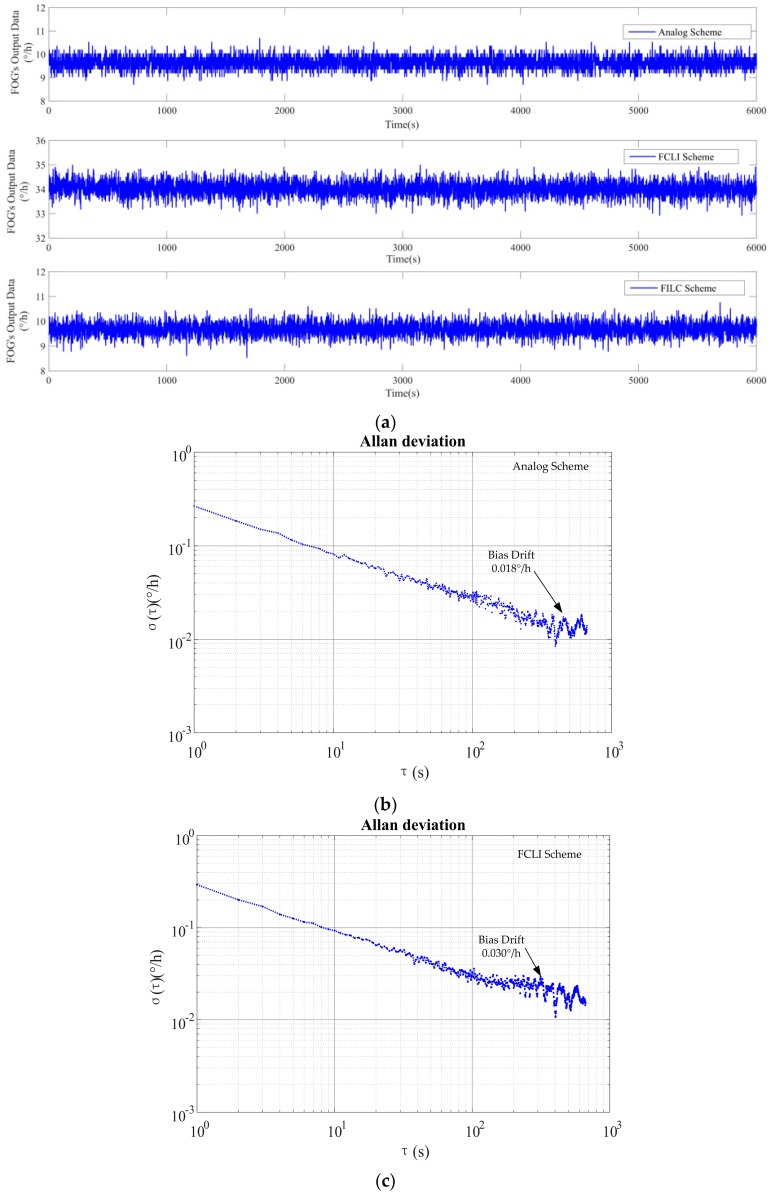

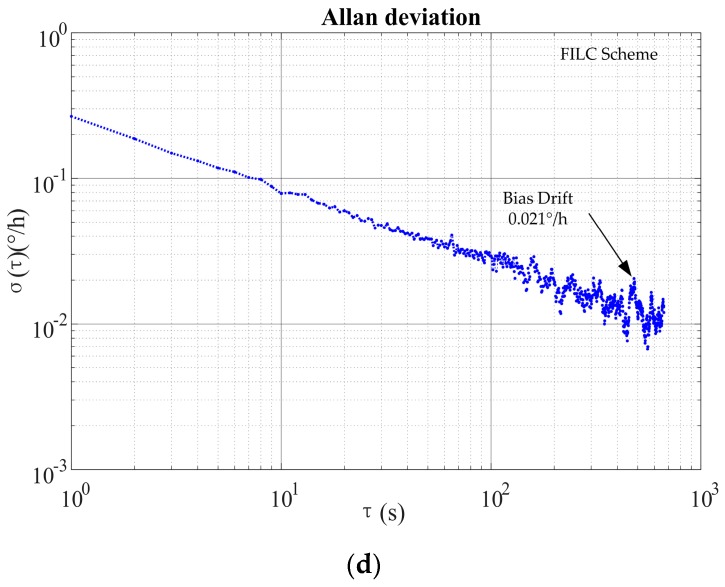
The raw output data and Allan deviations. (**a**) The raw output data of the three schemes; (**b**) the Allan deviation of the analog scheme; (**c**) the Allan deviation of the FCLI scheme; (**d**) the Allan deviation of the FILC scheme.

The raw output data with its corresponding Allan deviation for the three compensation schemes are shown in Figure 8, which is tested under the condition that the angular velocity is 9.6°/h and the scaling factor is approximately 0.60.

The bias performances of the three compensation methods are listed in Table 2.

In Table 2, the FILC scheme has a performance similar to the analog compensation scheme. Clearly, the bias of the FCLI scheme is larger than that of the other schemes. The noise of the FCLI scheme is 14% larger than that of the other methods, which is caused mainly by the quantization errors induced by the fluctuation of the scaling factor, as shown in Figure 7a, and partially by its bias drift, as shown in the Allan deviation plot in Figure 8c.

The parameters of the scale factor of those three different schemes are also tested as the input angular velocity increases from 0 to 300°/s with an increment of 10°/s. The scale factor is calculated by the least squares method according to [14]. In Table 3, the scale factors are nearly uniform in the three schemes, and the tiny differences are caused by gain fluctuation with residual quantization error in digital signal processing.

So far, we can fabricate high precision 0.01 °/h FOG without temperature compensation on bias. We believe our method is suitable for those FOGs without temperature compensation on bias.

## 5. Conclusions

A novel First-Integration-Last-Compression scheme is proposed to compensate the gain drift of the modulation chain in a fiber optic gyroscope. The FILC scheme guarantees the uniform time-average of the phase step between the modulation chain and the output chain and, consequently, suppresses the adverse effects of the quantization error on the output bias. Meanwhile, the FILC scheme keeps the advantage of automatic reset as an analog compensation scheme does, which simplifies the implementations of digital signal processing.

We verify the First-Integration-Last-Compression scheme on a middle-precision prototype of fiber optic gyroscope. The maximum residual quantization-induced bias achieves a value of 0.08 °/h over −40 °C to +60 °C, which is two times lower than the noise amplitude with data accumulated over one second sample intervals. The scale-factor linearity is 10 ppm. The results indicate that the FILC scheme is a feasible method to miniaturize FOG by integrating the function of the second feedback loop into the digital signal processor.

## Figures and Tables

**Table 1:** The designed parameters of the FOG.

Parameter	Value
L (length of fiber ring)	480 m
λ (wavelength of light)	1310 nm
D (diameter of fiber ring)	76.75 mm
n (bits of DAC)	16

**Table 2:** The bias parameters of three different schemes.

Scheme	Bias (°/h)	Noise (°/h)
the analog method	0.003	0.265
the FCLI scheme	23.620	0.303
the FILC scheme	0.043	0.266

**Table 3:** The parameters of scale factor of three different schemes.

Scheme	Scale Factor Bits (°/s)	Scale Factor Linearity Parts per Million (ppm)
the analog Scheme	43,800	8
the FCLI Scheme	43,831	17
the FILC Scheme	43,815	10
